# Heritable Gene Regulation in the CD4:CD8 T Cell Lineage Choice

**DOI:** 10.3389/fimmu.2017.00291

**Published:** 2017-03-22

**Authors:** Priya D. A. Issuree, Charles P. Ng, Dan R. Littman

**Affiliations:** ^1^The Kimmel Center for Biology and Medicine of the Skirball Institute, New York University School of Medicine, New York, NY, USA; ^2^Howard Hughes Medical Institute, New York University School of Medicine, New York, NY, USA

**Keywords:** DNA methyltransferase, ten eleven translocation enzymes, T cell development, gene silencing, RUNX3, Runx1, T helper inducing POZ/Krueppel-like factor, TCF transcription factors

## Abstract

The adaptive immune system is dependent on functionally distinct lineages of T cell antigen receptor αβ-expressing T cells that differentiate from a common progenitor in the thymus. CD4^+^CD8^+^ progenitor thymocytes undergo selection following interaction with MHC class I and class II molecules bearing peptide self-antigens, giving rise to CD8^+^ cytotoxic and CD4^+^ helper or regulatory T cell lineages, respectively. The strict correspondence of CD4 and CD8 expression with distinct cellular phenotypes has made their genes useful surrogates for investigating molecular mechanisms of lineage commitment. Studies of *Cd4* and *Cd8* transcriptional regulation have uncovered *cis-*regulatory elements that are critical for mediating epigenetic modifications at distinct stages of development to establish heritable transcriptional programs. In this review, we examine the epigenetic mechanisms involved in *Cd4* and *Cd8* gene regulation during T cell lineage specification and highlight the features that make this an attractive system for uncovering molecular mechanisms of heritability.

## Introduction

Conrad Waddington first coined the term “epigenetics” to refer to the study of the causal mechanisms connecting genotype with phenotype (1). It is well established that distinct cellular phenotypes in a multicellular organism arise from differences in gene regulation and not heterogeneity in DNA sequence. Gene expression patterns are preserved through cell division by heritable modifications of DNA and chromatin that expand the Watson–Crick base pairing information content of the genome. These adaptations comprise the “epigenetic landscape” crucial to cell lineage specification, depicted by Waddington as a marble rolling downhill into one of several furrows representing differentiated cell types (2). Early cytological studies distinguished heterochromatin that remained condensed throughout the cell cycle from euchromatin that had a diffuse appearance during interphase (3). Heterochromatin is generally tightly packed and transcriptionally quiescent. It can be further classified as constitutive heterochromatin composed of repetitive sequence elements such as telomeres and centromeres and facultative heterochromatin composed of genes that become silenced through developmental cues (4). In contrast, actively transcribed genes are typically located in accessible euchromatin. We are now beginning to understand the epigenetic processes that underlie heritable gene expression programs and characterize the physical properties of heterochromatin and euchromatin. Epigenetic mechanisms act on DNA and histones, both of which can be modified to regulate gene expression, such as by covalent histone linkages or methylation of cytosines in DNA [5-methyl cytosine (5mC)]. The combinatorial output of these marks provides a rich and diverse template for the development of distinct tissues and cell lineages, and in the era of genomics and computational biology, we are beginning to characterize the “epigenetic landscape” driving gene expression. In contrast to epigenetic changes that can occur during plastic stages of differentiation, the epigenetic programs controlling irreversible cell fate choices are key to understand the mechanisms of heritability. As we discuss below, in vertebrates, development of T cells expressing αβ T cell antigen receptors (TCRs) is a tractable system to study the epigenetic mechanisms of bi-potential cell fate decisions and the maintenance of gene expression states in differentiated cells. With ordered stages of maturation and defined molecular checkpoints at each stage, αβ T cell development provides an opportunity to study how chromatin changes drive gene expression during differentiation of a somatic tissue in adult animals. Furthermore, the study of CD4 and CD8, surface receptors whose expression corresponds to distinct T cell fates, has led to new insights into epigenetic inheritance.

## T Cell Development as a Model for Epigenetic Gene Regulation

T cell development begins when common lymphoid precursors from the bone marrow or fetal liver migrate through the blood to seed the thymus. Expression of the surface glycoproteins CD4 and CD8 distinguishes developmental stages of αβ T cells, with the most immature thymocytes being double negative (DN) for CD4 and CD8. Productive (in frame) VDJ recombination at the locus encoding the TCRβ chain is followed by assembly and signaling of the pre-TCR, which is composed of the beta chain paired with a germline-encoded pre-TCRα polypeptide. The signal induces robust proliferation and marks the passage of cells with productive rearrangements of their TCRβ chain genes, a process known as β-selection, to the next stage of development. This stage is characterized by upregulation of both CD4 and CD8, yielding double-positive (DP) thymocytes, and by rearrangement of the locus encoding the TCRα chain through VJ recombination. DP thymocytes that signal through sufficiently strong avidity interactions of their rearranged TCR with self-peptide/MHC molecules are positively selected, with CD4 and CD8 facilitating TCR signaling through their roles as co-receptors for MHC class II and MHC class I, respectively. Positive selection of cells with TCRs specific for MHC class II leads to the development of CD4^+^ single-positive (SP) thymocytes, composed of not only largely T helper cells but also some regulatory T cells with relatively high affinity TCRs. Conversely, thymocytes with TCRs that interact with MHC class I differentiate into CD8^+^ cytotoxic T cells. Meanwhile, cells with excessive TCR-MHC affinity are eliminated by negative selection to limit the release of autoreactive lymphocytes into the periphery.

There is a striking correspondence of co-receptor expression and commitment to functionally distinct lineages, indicating that regulation of CD4 and CD8 expression is linked to the functional programs of the developing T cells. This observation forms the basis of most models for lineage commitment, including the current kinetic signaling model (5, 6). The kinetic signaling model posits that as CD8 is downregulated following positive selection, leading to a CD4^+^CD8^lo^ phenotype, continued CD4 co-receptor expression allows for prolonged or stronger MHC class II-TCR signaling, inducing the helper T cell fate (7). Meanwhile, common gamma chain cytokines such as IL-7 rescue cells that have received an interrupted MHC class I signal and induce CD8 co-receptor reversal and the cytotoxic phenotype. “Top-down” studies of proximal TCR signaling have yielded little insight into how recognition of different types of MHC molecules results in distinct transcriptional programs. For this reason, and because of the intimate link between co-receptor expression and lineage commitment, “bottom-up” studies of *Cd4* and *Cd8* locus regulation have been undertaken as a way toward identifying signaling differences between the lineages. These studies have characterized an extensive transcriptional network that includes T helper inducing POZ/Krueppel-like factor (Thpok), Runx3, Mazr, Tcf1, and lymphoid enhancer factor 1 (Lef1) (8–10). Thpok and Runx3 are required for thymocytes to commit to the CD4 and CD8 lineages, respectively. Antagonistic cross-regulation between Thpok and Runx3 is essential to drive helper versus cytotoxic lineage choice, whereby Runx complexes limit the Thpok expression to MHC class II selected cells and Thpok represses Runx3 expression during differentiation toward CD4^+^ T cells. However, these transcription factors differ in their abilities to “redirect” cells so that they adopt the wrong fate following TCR–MHC interaction (11). Additional transcription factors also have important roles in lineage specification or the activation of lineage-specific genes, even if they do not directly control lineage commitment or repress genes of the wrong lineage (12). For example, GATA3 is required for the specification of thymocytes to the CD4 lineage, controlling expression of *Thpok*, and also participates with *Thpok* in “locking in” the lineage-specific program of gene expression.

Despite growing knowledge of the key transcription factors involved in lineage commitment, the mechanisms by which they direct cell fate decisions through epigenetic mechanisms to establish heritable programs of gene expression remain largely unknown. The study of the transcriptional regulation of the *Cd4* and *Cd8* loci, with their exquisite use of regulatory elements and key transcription factors to dictate temporal aspects of gene transcription, is slowly unraveling the orchestration of key epigenetic processes that subsequently allow for heritable gene expression patterns. As we discuss in this review, stage-specific *cis* elements at the *Cd4* locus have critical roles in establishing the epigenetic marks that allow for heritable transmission of gene states. This allows for a clear dissection of how these marks are deposited *via* transcription complexes and what epigenetic marks encode heritable information that is transmitted independently of these *cis* elements and transcription factors thereafter. In addition to being a tractable system whereby developmental stages can be easily followed, the *Cd4* and *Cd8* system also offers the potential to understand extracellular signaling cues that lead to the choreography of intricate epigenetic processes.

## Epigenetic Mechanisms of Heritable Gene Expression

### DNA Methylation

One of the best-studied epigenetic mechanisms of heritability is the covalent modification of cytosine to 5mC, a mark deposited by the DNA methyltransferase (DNMT) enzymes. DNA methylation occurs predominantly at cytosine residues that are followed by guanine (CpG) in mammalian genomes, and about 60–80% of CpGs are methylated in somatic tissues (13). The classic model of DNA methylation holds that *de novo* DNA methylation is deposited in the genome by Dnmt3a and Dnmt3b along with their non-enzymatic co-regulator Dnmt3L (14, 15). Maintenance DNA methylation is carried out by Dnmt1, which associates with the replication fork through PCNA and with hemimethylated CpGs through the E3 ubiquitin ligase Uhrf1 during DNA replication (16–18). However, these distinctions are not absolute as Dnmt1 has been shown to exhibit *de novo* methyltransferase function, and Dnmt3 can participate in the maintenance of methylation marks (19). Also, as discussed later, the model of DNA methylation was further revised with the discovery of an active enzymatic process of demethylation.

In the 1970s, two laboratories hypothesized that DNA methylation could act as a cellular mechanism of transcriptional memory through cell division due to the symmetrical nature of the CpG dinucleotide (20, 21). Since then, DNA methylation has been shown to be critical for genomic imprinting, X chromosome inactivation, and long-term repression of mobile genetic elements (22). Mechanistically, DNA methylation can lead to gene silencing by inhibiting the binding of factors that activate transcription through the addition of methyl groups in the major groove of the double helix or through the recruitment of repressive complexes (13). For example, the binding of CTCF, an insulator protein involved in the formation of chromosomal domains, is inhibited by DNA methylation, allowing enhancer-mediated activation of the paternal allele at the imprinted *H19/Igf2* locus (23). DNA methylation can also mediate gene repression through methyl-CpG-binding domain proteins that bind to 5mC. Some of these proteins such as Mbd2 and Mbd3 have been found to associate with the nucleosome remodeling and deacetylase (NuRD) complex (nucleosome remodeling and histone deacetylation), thus linking DNA methylation with other epigenetic mechanisms such as histone deacetylation and nucleosome positioning (24, 25). Functions beyond gene silencing have also been reported, such as genomic DNA methylation defining introns and exons during splicing (26, 27).

Although DNA methylation is a stable epigenetic mark that can be propagated across cell divisions, DNA demethylation had been observed in different biological contexts (28).

In contrast to passive demethylation, which results in the loss of 5mC during successive rounds of replication in the absence of DNA methylation maintenance machinery, replication-independent demethylation processes had also been observed invoking the possibility of active enzymatic removal of 5mC marks (29). The discovery of the ten eleven translocation (TET) family of enzymes that can modify 5mC through iterative oxidation converting 5mC to 5-hydroxymethyl cytosine (5hmC), and subsequently to 5-formylcytosine and 5-carboxylcytosine (5caC), and the detection of these intermediates *in vivo* in mammalian DNA, were critical in elucidating the mechanism of active DNA demethylation (30–32). There are three TET family members in mammals (Tet1/2/3), each possessing a core catalytic domain at the carboxyl terminus, a double-stranded β-helix fold containing crucial metal-binding residues, and a CpG DNA-binding CXXC domain toward the amino terminus of the protein (33). Although Tet1 and Tet3 contain an internal CXXC domain, Tet2 partners with IDAX, an independent CXXC-containing protein (34). Following iterative oxidation of 5mC to 5caC by the TET enzymes, unmodified cytosines can be generated *via* passive dilution of the oxidized base or enzymatic removal *via* thymine DNA glycosylase and the base excision repair pathway (35–38).

### Histone Modifications

In eukaryotes, DNA is wrapped around an octamer of histones composed of two copies of H2A, H2B, H3, and H4. The linker histone H1 is present where the DNA enters and exits the core nucleosome, and this nucleosome filament can be further compacted, facilitating the packing of large eukaryotic genomes within the limited space of the nucleus (39). Covalent changes in histones through posttranslational modifications (PTMs) are critical to epigenetic processes as they can alter chromatin structure or allow recruitment of activators or repressors of transcription (40). Examples of PTMs include acetylation, methylation, phosphorylation, ribosylation, and ubiquitination although it is unclear whether all of these are self-propagating through cell division, a typical criterion of epigenetic processes. One well-studied modification is histone acetylation at lysine residues mediated by histone acetyltransferases (HATs). Acetylation is typically associated with gene activation, whereas histone deacetylase enzymes (HDACs) that catalyze the hydrolysis of acetyl-lysine residues generally result in silencing and chromatin compaction (41–43). Histone methylation can also occur on lysine and arginine residues, and the methylation of distinct lysines on the histone H3 tail is associated with either gene silencing or activation. H3K9me2/3 is a conserved hallmark of heterochromatin among eukaryotes and serves as a binding site for heterochromatin protein 1 (HP1) family members to facilitate heterochromatin integrity (44). Furthermore, H3K9me3 also interacts with the DNA methylation machinery, and cooperation between these silencing marks may be vital for heritable silencing. For instance, Uhrf1 was found to bind H3K9me3 marks and facilitates H3 ubiquitination *via* its RING domain for recruitment of Dnmt1 at the replication fork (45–47). Deacetylation of histones can also provide H3 lysine substrates for the histone methyltransferase G9a, which also facilitates *de novo* DNA methylation at gene promoters through the recruitment of Dnmt3a and Dnmt3b in ES cells (48, 49). Interestingly, the histone methyltransferase activity of G9a is dispensable for *de novo* methylation at G9a-target gene promoters in ES cells (50). A more direct connection between DNA methylation and H3K9 methylation exists in the fungus *Neurospora crassa* in which the H3 histone methyltransferase *dim-5* is required for DNA methylation through trimethylation of H3K9 (51). Other repressive histone marks include H3K27me2/3, mediated by the polycomb group 2 complex (PRC2), modifications associated with the silencing of genes during developmental decisions (52, 53). In contrast to K9/K27 methylation, H3K4 trimethylation (H3K4me3) is associated with active promoters, whereas H3K4 monomethylation (H3K4me1) is often a mark of enhancer function (54). H3K4me3 was also suggested to inhibit DNA methylation, as the *de novo* DNA methylation co-factor Dnmt3L was blocked from binding methylated H3K4 (55). Several histone modifications have also been found to be reversible by enzymes such as the histone demethylase Lsd1 that removes monoethyl or dimethyl marks from H3K4 (H3K4me1/2) (44).

Non-covalent histone modifications can also impact the state of chromatin through changes in nucleosome positioning or altered exposure of DNA along the nucleosome through ATP-dependent nucleosome remodelers. Indeed, nucleosome remodelers are critical for chromatin assembly and enable dynamic changes in genomic architecture in response to signaling or differentiation (56). Many of these complexes can both activate and repress transcription, possibly through combinatorial assembly of different subunits (57). Their specificity is thought to stem from recruitment by histone modifications, transcription factors, or DNA methylation, and therefore they have an intimate connection to epigenetic processes. Thus, both covalent and non-covalent histone modifications extend the capacity of chromatin to influence gene expression.

### Histone Variants

Variants of the core histones H2A, H2B, and H3 and the linker histone H1 exist and differ from their canonical histone counterparts in primary sequence and expression pattern. In contrast to S phase-coupled expression of the canonical histones, histone variants are expressed throughout the cell cycle (39). Substitution of canonical histones with these variants is thought to modulate gene expression by altering the physical properties of nucleosomes through changes in PTMs, chromatin structure, or recruitment of additional co-activators or repressors (58). Some examples of histone variants that have a putative role in transcription include the H2A variant H2A.Z, which is enriched at the transcription start site (TSS) of active genes, and the H3.1/H3.2 variant H3.3, which has been suggested to have roles in both gene activation and heterochromatin integrity (59, 60).

### Other Modes of Epigenetic Regulation

Although epigenetic marks are required for proper regulation of gene expression, an area of active interest is the contribution of small and long non-coding RNAs (ncRNAs) in directing chromatin modifications. In yeast, plants, and *Drosophila*, a role for small ncRNAs has been implicated in heterochromatin formation, which is disrupted upon interference with the RNAi machinery (61–63). Whether a similar pathway operates in mammals is still unclear as studies using genetic knockout of the endoribonuclease Dicer in ES cells, which is required for the biogenesis of siRNAs and microRNAs, resulted in different conclusions, with one study showing no effect on histone modification or DNA methylation status (64, 65). The PIWI-interacting RNA (piRNA) pathway has also been demonstrated to regulate heterochromatin in *Drosophila* by directing H3K9 methylation to transcriptionally silence transposons and interacting with HP1 (66, 67). In mice, the piRNA pathway seems to be required for sequence specific *de novo* methylation of the imprinted *Rasgrf1* locus in the male germline (68). The epigenetic landscape can also be shaped *via* site-specific chromatin modifications mediated by long ncRNAs (lncRNAs). To date, X chromosome inactivation is the canonical model for the epigenetic regulation by lncRNAs. The X-inactive-specific transcript (*Xist*) is transcribed from the inactive X chromosome in female mammalian cells and has been shown to mediate silencing of the X chromosome in *cis* through the direct recruitment of PRC2 and YY1 (69, 70). Since the discovery of *Xist*, additional lncRNAs have been implicated in the modulation of epigenetic processes at other loci through interaction with chromatin modifiers (71). However, the *in vivo* functions of many lncRNAs have not yet been evaluated.

**Figure 1:**
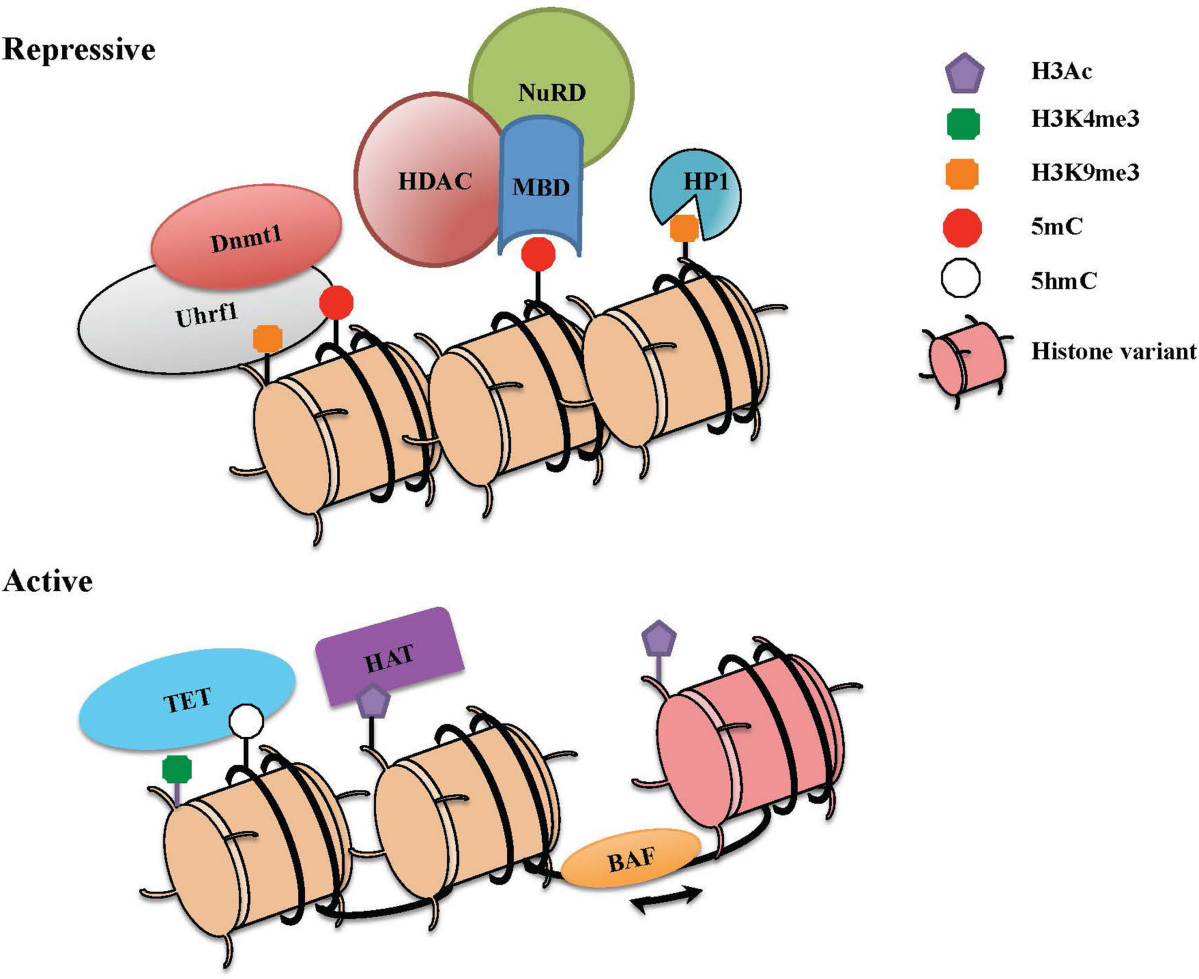
**The combinatorial readout of chromatin dictates repressive versus active states of gene expression**. Chromatin is a dynamic structure that enables gene regulation and packaging of the eukaryotic genome into the nucleus. The combinatorial output of chromatin modifications, including covalent histone linkages, histone variants, nucleosome occupancy, and DNA methylation patterns, dictate gene expression patterns in distinct tissues and cell lineages. (Top) Heterochromatin depicts a condensed and transcriptionally silent conformation of DNA. Silent genes in heterochromatin contain histone marks such as H3K9Me2/3 that can be bound by heterochromatin protein 1 (HP1), associates with DNA methylation machinery, and also recruits repressive complexes. High levels of DNA methylation are maintained through the action of Dnmt1 and its partner Uhrf1, which also interacts with H3K9me. Methyl-CpG-binding proteins that specifically recognize methylated DNA can recruit the nucleosome remodeling and deacetylase (NuRD) complex, which partakes in chromatin remodeling and catalyzes histone deacetylation though histone deacetylase enzymes (HDACs). (Bottom) To activate previously silent genes, activating transcription factors can recruit histone acetyltransferases (HATs) and chromatin remodeling complexes (BAF) to promoters and enhancers. Nucleosome remodeling can allow for transcription through nucleosome sliding and increased accessibility, exchange of a standard histone for a histone variant such as H2A.Z or eviction of the nucleosome. The DNA demethylase enzymes [ten eleven translocations (TETs)] also catalyze the conversion of methylcytosine to 5-hydroxymethylcytosine (5hmC) and other oxidation products, which in turn can inhibit the binding of repressive complexes and promote gene expression.

Taken together, epigenetic changes are dictated by numerous mechanisms that work in a combinatorial manner (Figure 1). An understanding of how these processes work in combination and how they are deployed for heritable transmission of gene expression has proven challenging. As we review below, studies of *Cd4* and *Cd8* regulation during lineage commitment may help to shape our basic understanding of these processes in a system that is highly tractable.

## *cis* Elements and Epigenetic Regulation of the *Cd4* Locus

**Figure 2:**
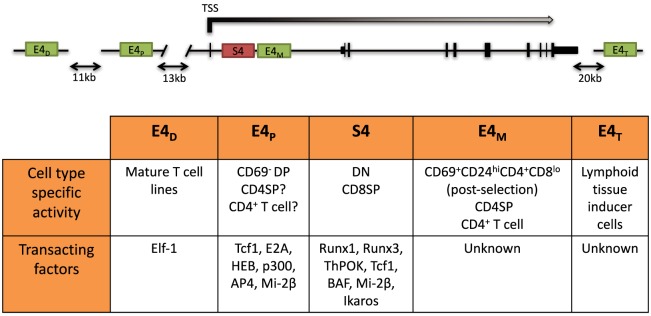
**Schematic of the *Cd4* locus and its *cis*-regulatory elements**. (Top) Two positive *cis* elements, E4_P_ and E4_M_, and a silencer element, S4, regulate CD4 expression in αβ T cells. E4_P_, consisting of a 339 bp DNA element, lies ~13 kb upstream from the *Cd4* transcription start site (TSS) and is required for *Cd4* expression in double-positive (DP) thymocytes and establishment of stable CD4 expression following positive selection of CD4 lineage cells. The 434 bp S4 element lies ~1.5 kb downstream of the *Cd4* TSS in the first intron and represses *Cd4* expression in double-negative (DN) and cytotoxic CD8^+^ T cells. E4_M_ is immediately downstream of S4 and promotes *Cd4* expression in postselected CD4 thymocytes and mature CD4^+^ T cells. The distal enhancer E4_D_ was shown to promote CD4 reporter gene expression in T cell lines but was not assessed *in vivo*. The thymocyte enhancer E4_T_ was shown to direct CD4 expression in lymphoid tissue inducer cells by mutation of the endogenous locus. (Bottom) Table of the cell type-specific activity of the different *cis* elements and known transacting factors reported to bind and modulate expression of *Cd4* (description in text).

The dynamic regulation of the CD4 co-receptor during αβT cell development is finely controlled *via* multiple *cis*-regulatory elements that were initially revealed by functionally testing DNase I hypersensitivity sites (DHSs). Four *cis* elements were reported to direct *Cd4* expression in transgenic assays: a silencer (S4) situated 1 kb downstream of the *Cd4* TSS, a proximal enhancer (PE) (E4_P_), a distal enhancer (E4_D_) situated 13 and 24 kb upstream of the *Cd4* TSS, and a thymocyte enhancer (E4_T_) mapped 36 kb downstream. The *in vivo* roles of only three have thus far been assessed by targeted deletion of the endogenous locus. E4_P_ and S4 have been found to direct expression of *Cd4* in αβ T cells, and the thymocyte enhancer E4_T_ was found to direct *Cd4* expression in lymphoid tissu inducer (LTi) cells in the intestine (72, 73). Although E4_D_ was reported to drive reporter expression in mature T cell lines, its function still remains to be assessed *in vivo* (74). Recently, an intronic enhancer adjacent to S4, ~2 kb downstream of the *Cd4* TSS, which we refer to as E4_M_, was found to drive CD4 expression in αβ T cells and is described further below (Figure 2) (75).

### E4_P_

The E4_P_ element was mapped to a 339 bp region of DNA that directed reporter activity in T cell lines and *in vivo* in T cells of transgenic reporter mice (76, 77). Germline targeting of E4_P_ prevented CD4 upregulation during the DN–DP transition in the thymus, demonstrating its requirement for CD4 expression in preselection DP cells (73). After positive selection, CD4 was reexpressed on MHC class II selected cells, although at reduced levels compared to wild-type cells. Furthermore, CD4 expression was unstable during cell division, as a large proportion of CD4^+^ T cells in the periphery lost CD4 expression after activation. In these cells, H3 acetylation and H3K4Me3 within the *Cd4* locus were also reduced. The loss of H3Ac and H3K4Me3 was observed in CD4^−^ DP stage thymocytes, consistent with the loss of CD4 expression. To investigate whether E4_P_ was required for regulating CD4 expression beyond the DP stage, Cre-mediated excision of a conditional allele was performed. In contrast to germline deletion, conditional deletion of E4_P_ in mature CD4^+^ T cells did not impair CD4 expression during activation, and the levels of H3Ac and H3K4Me3 were similar to wild-type cells. This suggested that, during thymic development, E4_P_ modulates a stable epigenetic state of the *Cd4* locus that can be maintained in its absence thereafter.

Sequence analysis and nuclear extract binding studies suggested that the HMG-box transcription factors T cell factor 1 (Tcf1)/Lef1 and the basic helix-loop-helix (bHLH) proteins E2A/HEB bind to motifs within E4_P_ (76, 78). Accordingly, gene targeted inactivation of the genes encoding E2A and HEB impaired CD4 expression during the DN–DP transition (79, 80). Similarly, mutation of *Tcf7* (which encodes Tcf1) also reduced CD4 expression in DP thymocytes (81). ChIP analyses confirmed that these factors bound to E4_P_, suggesting that they directly promote expression of CD4 in DP thymocytes (81, 82). However none of these factors is individually required for CD4 expression in DP thymocytes, as single deletions do not phenocopy E4_P_^−/−^ mice. Interestingly, the NuRD complex, typically associated with gene repression, was also implicated in CD4 activation (83). Deficiency in mi-2β, an ATPase chromatin remodeling subunit of the NuRD complex, led to impaired CD4 upregulation during the DN–DP transition (83). mi-2β also bound E4_P_ by ChIP assays and co-immunoprecipitated with HEB and the HAT p300, linking histone acetylation with factors recruited to E4_P_. The cooperative mechanisms by which these transcription factors drive transcription of *Cd4* and their contribution to epigenetic programs are yet to be assessed.

### E4_M_

The observation that CD4 was re-expressed following positive selection in E4_P_^−/−^ thymocytes suggested the existence of another regulatory element, now referred to as the “maturation enhancer,” E4_M_. The existence of such an element was also supported by a previous study employing a *Cd4* transgene containing the *Cd4* first intron, promoter, and E4_P_ element, whereby transgenic reporter activity was lost in activated mature T cells without changes in endogenous *Cd4* expression (84). Indeed, our recent ATAC-seq analysis of the *Cd4* locus in CD4SP thymocytes revealed a previously uncharacterized chromatin-accessible region situated in the first intron of the gene, 3′ to the silencer (Priya D. A. Issuree and Dan R. Littman, unpublished results). The potential role of this region was highlighted by Egawa and colleagues, who showed that CD4 expression was unstable in T cells from mice with targeted deletion of 1.5 kb encompassing the silencer and the region 3′ to it, but was stable when only the core silencer was deleted (75). Our studies using mice lacking E4_M_ revealed that this element controls *Cd4* expression in postselected CD4SP thymocytes, and in the absence of both E4_M_ and E4_P_, there is a complete loss of CD4 in T cells (Priya D. A. Issuree and Dan R. Littman, unpublished results). As observed in the absence of E4_P_, deletion of E4_M_ resulted in the gradual loss of CD4 expression by activated CD4^+^ T cells following multiple rounds of cell division [(75) Priya D. A. Issuree and Dan R. Littman, unpublished results]. Analogous to E4_P_, retroviral Cre-mediated excision of this region in mature T cells did not diminish CD4 expression, suggesting that E4_M_ may be required for establishing an epigenetically active state during development. However, it remains possible that both enhancers function cooperatively in the establishment of stable CD4 expression and are insufficient to do so individually. Importantly, these studies demonstrate that the main enhancers dictating CD4 expression in αβ T cells are E4_P_ and E4_M_, and no additional elements are capable of compensating for their loss. The minimal region encompassing E4_M_ activity and the transacting factors recruited there to drive expression are currently under investigation. It also remains to be determined whether the lack of E4_M_ without a larger intronic deletion results in redirection of MHC class II selected cells into the CD8^+^ lineage, as was observed in mice with the 1.5-kb intronic deletion (75).

### *Cd4* Silencer (S4)

The silencer (S4) is a 434 bp core element in the first intron of *Cd4* that was identified by its ability to inhibit expression from reporter constructs in cell lines and in both DN thymocytes and CD8^+^ T cells of transgenic mice (77, 85). Deletion of this element in the germline of mice caused CD4 derepression in DN thymocytes and CD8^+^ cytotoxic T cells, confirming its physiological function (86, 87). Importantly, Cre-mediated excision of a conditional LoxP-flanked S4 allele in mature CD8^+^ T cells did not reverse CD4 repression (87). The results demonstrated that S4 activity is reversible during the DN–DP transition, but becomes irreversible following positive selection, in mature cytotoxic T cells. Therefore, CD4 silencing in peripheral cytotoxic T cells is independent of S4 and mediated by a heritable silencing mechanism after CD8^+^ lineage commitment. To determine when S4-independent repression occurred, stage-specific S4 deletion with different Cre transgenic mice was carried out. By using CD4-Cre, which drives Cre expression in the late DN and early DP stages of development, DN thymocyte repression was intact but mature CD8^+^ T cells derepressed CD4 similarly to the germline deletion (87). S4 deletion with CD8-E8_I_ Cre, which is active only in MHC class I-selected T cells, also caused CD4 derepression, suggesting that heritable *Cd4* silencing occurs after positive selection. Deletion of S4 at additional stages after MHC class I-positive selection may provide more insight into the initiation of heritable CD4 silencing.

**Figure 3:**
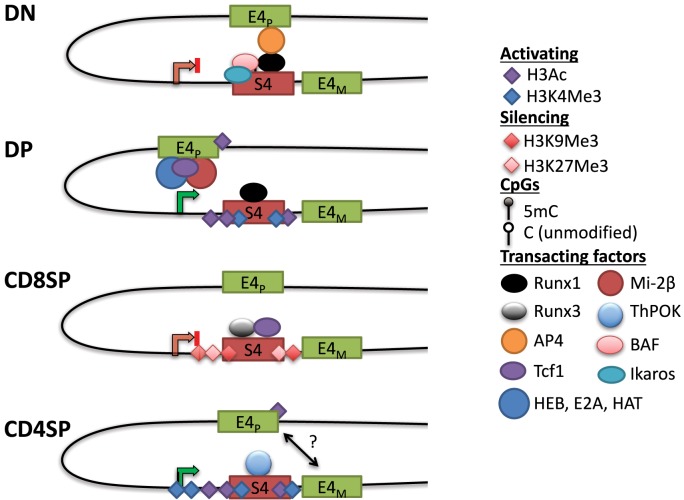
**Dynamic *Cd4* regulation during T cell development in the thymus**. In double-negative (DN) thymocytes, *Cd4* expression is repressed through S4, which may interact with the E4p enhancer, potentially preventing it from activating the *Cd4* promoter. This interaction could be mediated *via* Runx1, which binds to S4 and associates with AP4 at E4_P_, possibly through chromatin looping (see text). Nucleosome remodeling complexes such as the SWI/SNF-like Brg/Brahma-associated factor (BAF) complex are thought to regulate accessibility to S4 to promote silencing. The *Cd4* locus in DN thymocytes contains high levels of DNA methylation and lacks high levels of histone acetylation. In double-positive (DP) thymocytes, *Cd4* expression is upregulated through E4_P_, potentially due to changes in conformation of the locus (see text). E4_P_ serves as the recruitment site for numerous transcription factors including HEB, E2A, Tcf1, and lymphoid enhancer factor 1. The ATPase subunit of the nucleosome remodeling and deacetylase complex, mi-2β, is a positive regulator of *Cd4* expression, possibly through the recruitment of histone acetyltransferases (HATs) to either E4_P_ or S4. The locus acquires H3Ac and H3K4 trimethylation (H3K4Me3) at S4, but DNA methylation remains high (see Figure 4). In CD8SP cells, *Cd4* expression is repressed through the transcription factor Runx3 and Tcf1 binding to S4. The locus acquires some additional DNA methylation and silencing histone marks H3K9Me3 and H3K27Me3. In CD4SP cells, E4_M_ controls *Cd4* potentially in a cooperative manner with E4_P_. S4 is bound by the CD4^+^ lineage commitment factor T helper inducing POZ/Krueppel-like factor (Thpok), which antagonizes activity of Runx complexes. The locus undergoes active demethylation and is thus hypomethylated in CD4SP cells relative to all other stages of T cell development (see Figure 4).

In contrast to uniform CD4 derepression in S4^−/−^ mice, variegated CD4 derepression occurred with smaller deletions in S4 (88). This phenotype was reminiscent of position-effect variegation (PEV) studied extensively in *Drosophila*. PEV occurs when a gene is stochastically silenced in a population of cells due to spreading of heterochromatin from an adjacent locus (89). In agreement with the spreading of heterochromatin, CD4 repression was stable in cells that achieved silencing and the frequency of repression could be increased by overexpressing HP1β *in vivo* (88). There was no variegation observed in DN cells in which CD4 derepression occurred following mutations in S4. Instead, there was a partial but uniform derepression, consistent with active repression and the contribution of multiple transcription factor binding sites toward this process (88). Motif analysis revealed two sites in S4 that contained consensus binding motifs for members of the evolutionarily conserved family of Runx transcription factors (see Runx) (90). Deletion of both sites caused CD4 derepression in CD8^+^ T cells, and interaction between S4 and the Runt domain of Runx1 was found in a yeast-one-hybrid screen (91). Runx1 is expressed at the highest level in DN thymocytes, whereas Runx3 is predominantly expressed in CD8SP cells. Consistent with their expression pattern, loss of function studies demonstrated that Runx1 is indispensable for active repression of *Cd4* in DN thymocytes, whereas Runx3 is responsible for establishing *Cd4* silencing in CD8^+^ T cells (91–94). Interestingly, deficiency in the nucleosome remodeling Brg/Brahma-associated factor (BAF) complex was also reported to cause CD4 derepression in DN thymocytes (82). Combining BAF deficiency with point mutants in S4 significantly enhanced CD4 derepression, and BAF subunits were found to bind S4 (82). The BAF complex was subsequently implicated in regulating S4 accessibility for Runx1 binding in DN thymocytes (95). Finally, the zinc finger transcription factor Ikaros, which associates with BAF and other chromatin remodeling complexes, was also found to be required for CD4 repression in DN thymocytes (96). Active repression of CD4 hence appears to require remodeling of the locus and binding of Runx1, which is discussed further below (Figure 3).

S4 deletion increased recruitment of HAT p300 at E4_P_ and increased H3K9Ac at the promoter in DN thymocytes (93). Although E4_P_ is 13 kb upstream of the *Cd4* promoter, these elements have been suggested to physically communicate through looping (97). Chromatin conformation capture assays suggested that in DN thymocytes, Runx1 mediates a loop between S4 and E4_P_, which sequesters the positive transcription elongation factor (P-TEFb) from activating RNAPol-II at the promoter (97). Another factor that cooperates with Runx1 in *Cd4* silencing at the DN stage is the bHLH transcription factor adaptor-related protein complex 4 (AP4), which binds to E4_P_ (98). This raises the possibility that a loop forming between E4_P_ and S4 *via* Runx1 and AP4 prevents transcription in DN thymocytes and, upon Runx1 downregulation during the DN–DP transition, P-TEFb associates with RNAPol-II at the promoter to drive expression. However, Runx1 has also been reported to bind to S4 in DP thymocytes, suggesting that its repressive function is overridden by an unknown mechanism at this stage. Another study using three-dimensional fluorescent *in situ* hybridization showed that the *Cd4* and *Cd8* loci are in close proximity when CD8 is expressed (in DP thymocytes and CD8^+^ T cells), but in the absence of S4, this association was lost, suggesting that S4 has a role in locus architecture (99). Strikingly, association of the co-receptor loci was conserved in human T cells even though *Cd4* and *Cd8* are on different chromosomes, suggesting that their proximity may be functionally important. Thus, S4 together with E4_P_ and E4_M_ are able to direct helper lineage-specific expression of CD4 *in vivo*.

Taken together, the study of *cis* elements controlling *Cd4* expression during T cell development revealed a key role for these elements in the establishment of key epigenetic marks that can be transmitted thereafter in a heritable manner. Thus, a thorough analysis of the stage-specific activity of these elements provides a unique opportunity to delve into molecular requirements for heritable transmission.

## DNA Methylation as a Key Epigenetic Mark for the Heritable Expression of *Cd4*

The nature of the epigenetic process employed to enable heritable silencing of *Cd4* in mature CD8 T cells in the absence of S4 was a missing piece in our understanding until recently. An shRNA screen, designed to identify genes repressing *Cd4* in mature CD8^+^ T cells independently of S4, led to the identification of Dnmt1 and the DNA methylation pathway as a central requirement to this process (100). Although previous experiments had concluded that DNA methylation was not involved in CD4 silencing, these results were obtained with 5-azacytidine, a potent inhibitor of DNA methylation, that likely obscured CD4 derepression due to toxicity in primary CD8^+^ T cells (87). Indeed, a subsequent study with reduced dosing of the inhibitor showed CD4 derepression (75). Additional genetic evidence demonstrating the role of DNA methylation in CD4 silencing was shown through the use of mice with mutations in Dnmt1 and the *de novo* DNMTs, Dnmt3a and Dnmt3b. Transfer of DNMT-deficient naïve CD8^+^ T cells into lymphopenic mice resulted in robust CD4 derepression following homeostatic proliferation. Interestingly CD4 expression in naïve T cells from Dnmt-deficient mice is normal, suggesting that CD4 derepression in the absence of DNA methylation machinery occurs during robust T cell proliferation. Whether the conditional inactivation of the Dnmt3 enzymes using CD4-Cre influences CD4 derepression due to effects on *de novo* rather than maintenance methylation remains to be determined.

The changes in DNA methylation at the *Cd4* locus during thymic development were assessed using bisulfite CATCH-seq (clone-adapted template-capture-hybridization sequencing). A region displaying a high level of differential methylation [differentially methylated region (DMR)] between CD4^+^ and CD8^+^ T cells was identified in the first intron, extending from −0.7 kb to +3.2 kb relative to the *Cd4* transcriptional start site. The levels of DNA methylation during development correlated with CD4 expression, such that the locus was hypermethylated in DN and CD8SP thymocytes compared to CD4SP thymocytes. Surprisingly, CD4 expression in DP thymocytes was uncoupled from DNA methylation, as the *Cd4* locus was hypermethylated and resembled methylation patterns in CD8SP cells. However, a few additional methylated CpGs were found in CD8SP thymocytes compared to DP thymocytes, presumably due to the activity of Dnmt3 enzymes. It is possible that these methylated CpGs are sufficient to repress *Cd4* transcription, for example, by influencing recruitment of activating or repressive transcription factors to the locus or decreasing chromatin accessibility through nucleosome remodeling. As bisulfite sequencing does not discriminate between 5mC and 5hmC, another possibility is that some of the methylated CpGs at the *Cd4* locus are 5hmCs, whose presence would promote CD4 expression (101). It is also possible that additional chromatin modifications induced by S4 through lineage-specific transcription factors such as Runx3 may be required to initiate CD4 repression during commitment to the cytotoxic lineage. Analysis of the *Cd4* locus revealed that the hypermethylation status of the DMR in CD8^+^ T cells was critically dependent on S4. In the absence of S4, the *Cd4* DMR methylation levels in CD4^+^CD8^+^ cytotoxic T cells resembled those in WT CD4^+^ T cells. Thus, S4 may be required to prevent demethylation after commitment to the CD8^+^ lineage and to participate in the establishment of heritable methylation marks for stable repression of CD4 in CD8^+^ T cells. Future investigation will allow testing of these hypotheses and address the contribution of factors involved in *Cd4* silencing such as Runx3 and Tcf1. Taken together, the results indicate that DNA methylation mediated by S4 is required, at least in part, for heritable silencing in the cytotoxic lineage.

## A Role for TET-Mediated DNA Demethylation in Heritable Expression of CD4

**Figure 4:**
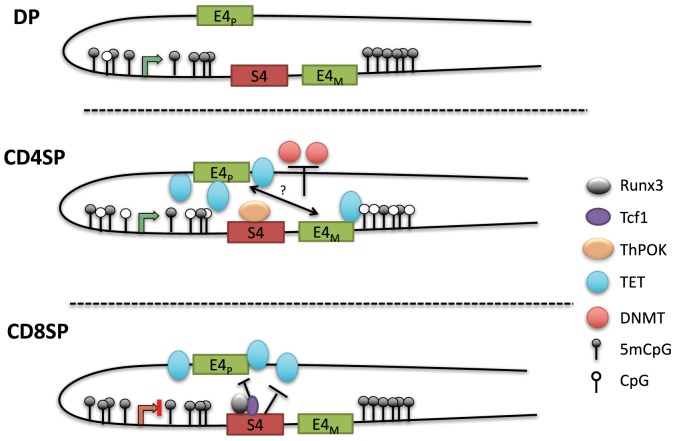
**Model of methylation dynamics at the *Cd4* locus during lineage commitment**. The *Cd4* locus is hypermethylated in double-positive (DP) thymocytes; as MHC class II selected cells downregulate CD8 to become CD4SP cells, the locus undergoes active demethylation presumably through iterative oxidation *via* ten eleven translocation (TET) enzymes. T helper inducing POZ/Krueppel-like factor (Thpok), which binds to S4 late during positive selection, partially contributes to the demethylation process. In the absence of E4_P_, the locus remains hypermethylated in CD4SP cells and resembles the methylation profile of CD8SP cells. Thus, E4_P_ mediates demethylation of the locus, perhaps in cooperation with E4_M_ following the selection by MHC class II molecules. In CD8SP cells, which undergo selection for MHC class I interaction, the *Cd4* locus remains hypermethylated (with addition of a few novel methyl-CpGs). However, in the absence of S4, the locus undergoes demethylation similarly to CD4SP thymocytes, indicating that S4 potentially inhibits *Cd4* demethylation by antagonizing ten eleven translocation (TET)-mediated demethylation.

The loss of methylation initially present in DN and DP cells during CD4^+^ T cell differentiation suggested that DNA demethylation is crucial for maintaining CD4 expression (100). As there is a lack of cell division during the DP to SP transition following positive selection, the decreased methylation in CD4SP cells suggested an active demethylation process rather than the lack of methylation maintenance during replication. Indeed, locus-specific oxidative bisulfite amplicon sequencing detected the presence of 5hmC at the *Cd4* locus in CD4^+^CD8^lo^ thymocytes as they differentiated into CD4SP cells. A subsequent study also found that 5hmC levels were correlated with gene activation in the thymus, and 5hmC was enriched in genomic regions harboring H3K4Me1 and H3K27Ac, marks associated with enhancers (102). The precise functions of the three TET enzymes in lineage commitment, and the mechanism of their recruitment to the *Cd4* locus are not known. Remarkably, although S4 was associated with increased methylation in CD8SP cells, the PE E4_P_ was critical for demethylation in CD4SP cells (Figure 4). E4_P_^−/−^ naïve CD4^+^ T cells exhibited hypermethylation of the *Cd4* DMR similar to WT CD8^+^ T cells. Furthermore, the loss of CD4 expression in activated E4_P_^−/−^ helper T cells correlated with increased DNA methylation of the locus. Thus, E4_P_ is required during development for the establishment of a heritable hypomethylated state at the *Cd4* locus in mature CD4^+^ T cells. It will be interesting to determine how E4_P_ coordinates DNA demethylation of the locus. In addition, in the absence of Thpok, the *Cd4* locus was hypomethylated in MHC class II selected cells redirected to the CD8^+^ lineage compared to WT CD8^+^ lineage cells, suggesting that Thpok is partially dispensable for DNA demethylation (100). Taken together, these results reveal a critical role for *cis*-regulatory elements in coordinating the proper epigenetic machinery during development that is essential for heritable *Cd4* expression (Figure 4). Elucidation of how transcription factors binding to these elements choreograph the functions of Dnmts and TET enzymes promises to reveal important aspects of differential signaling in positive selection of CD4 and CD8 SP cells.

## *cis* Elements and Epigenetic Regulation of the *Cd8* Locus

### CD8 Enhancers

**Figure 5:**
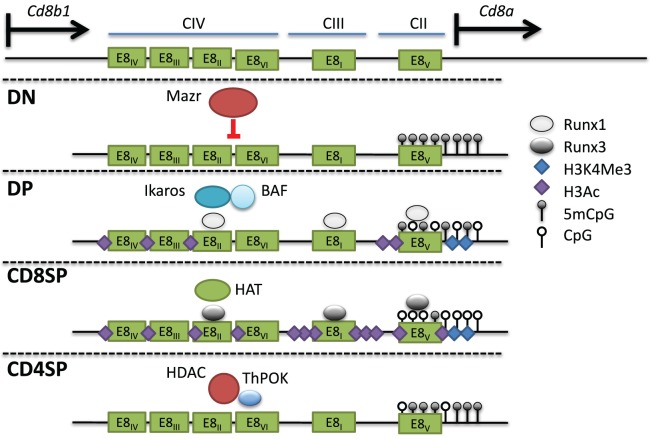
**Multiple *cis* elements direct *Cd8* expression during thymocyte development**. (Top) The *Cd8* locus consists of two linked genes, *Cd8a* and *Cd8b1*, on mouse chromosome 6. *Cd8* expression is regulated by at least six enhancers (E8_I-VI_) found in three clusters of DNAse I hypersensitivity sites. In double-negative (DN) thymocytes, Mazr represses expression of *Cd8*, and the locus is hypermethylated and lacks activating histone marks. Following pre-T cell antigen receptor signaling and maturation to become double-positive (DP) thymocytes, *Cd8* is upregulated *via* the control of multiple enhancers (see text). The chromatin remodeling Brg/Brahma-associated factor (BAF) complex and the transcription factor Ikaros promote *Cd8* expression in DP thymocytes. Runx1 also binds to multiple *Cd8* enhancers and was shown to promote *Cd8* expression as well. The locus in DP thymocytes partially loses DNA methylation and gains H3Ac and H3K4Me3 at the *Cd8a* promoter. In CD8SP cells, Runx3 binds to numerous enhancers leading to a corresponding increase in H3Ac, possibly due to direct recruitment of histone acetyltransferases (HATs), as well as a decrease in DNA methylation. In CD4SP cells, T helper inducing POZ/Krueppel-like factor recruits histone deacetylase enzyme to actively repress expression.

The *Cd8* locus is composed of two linked genes, *Cd8a* and *Cd8b1*. In mice, they are found on chromosome 6, separated by 36 kb and aligned in the same transcriptional orientation. DP thymocytes and most cytotoxic TCRαβ T cells express CD8 as a CD8αβ heterodimer, whereas on intraepithelial lymphocytes (IELs) and a subset of dendritic cells, CD8 can be expressed as a homodimer of CD8αα. This suggests that both genes can be coordinately and independently regulated in different cell types. An 80-kb genomic fragment encompassing the *Cd8* locus was found to drive developmental stage and lineage-specific expression of a reporter transgene (103, 104). Four DHS clusters within this fragment (CI-IV) contain at least six enhancers. E8_I_ enhancer (CIII-1,2) was found to drive CD8 expression in CD8SP thymocytes, mature CD8^+^ T cells, and IELs (104–106). Interestingly, the onset of E8_I_ activity was observed in positively selected thymocytes, suggesting a link between this element and cytotoxic T cell commitment. E8_II_ (CIV-4,5) directed expression in both DP thymocytes and CD8^+^ T cells, while E8_III_ (CIV-3) drove CD8 expression in immature DP thymocytes. The E8_IV_ (CIV-1,2) element was active in DP and CD8^+^ T cells. In transgenic studies, E8_V_ (CII) exhibited no enhancer function by itself, but a combination of E8_V_ and E8_I_ directed expression in both CD8^+^ T cells and DP thymocytes, unlike E8_I_ alone, suggesting that stage-specific activity could arise through combinations of CD8 enhancers (107). Recently, another *cis* element termed E8_VI_ (CIV-6) was described, which directed transgenic reporter expression in mature CD8^+^ T cells, memory-phenotype-like CD44^hi^CD62L^+^CD8^+^ T cells, and CD8αα^+^ dendritic cells (108). In summary, at least six enhancers direct CD8 expression in cytotoxic T cells (Figure 5).

Studies examining the regulation of the endogenous *Cd8* locus through genetic deletion revealed both specific and cooperative modes of enhancer regulation. In E8_I_^−/−^ mice, both TCRγδ and TCRαβ IELs showed a significant decrease in CD8α, while CD8 expression in DP thymocytes, CD8SP thymocytes, and peripheral CD8^+^ T cells was largely intact (106, 107). E8_I_ was also found to be crucial for the maintenance of CD8α expression in activated CD8^+^ T cells (109). In some cases, combinatorial enhancer activity occurred as shown by combined deletion of E8_I_/E8_II_, resulting in variegated expression in DP thymocytes that did not occur with single deletion of either enhancer (110). In contrast, combined deletion of E8_I_/E8_II_ had no additional effect on the maintenance of CD8 expression in activated CD8^+^ T cells than deletion of E8_I_ alone (109). These results suggested that E8_I_ might have a non-redundant role in maintaining transcription of CD8α during activation, as IELs are also thought to exist in a partially activated state (111). Similar to combined E8_I_/E8_II_ deletion, variegated CD8 expression was observed during the DN–DP transition with deletion of E8_V_ or combined deletion of E8_II_/E8_III_ (112, 113). Taken together, these data indicate that *in vivo* the CD8 enhancers have both non-redundant and stage-specific functions.

### Search for a CD8 Silencer Element

It is unclear whether there is a silencer in the *Cd8* locus, analogous to that in *Cd4*, that restricts its expression to the cytotoxic lineage. Experiments in a hybridoma cell line suggested the possibility that DNA elements in *cis* to the *Cd8* locus were able to silence transcription (114). Referred to as L2a, this region within E8_V_ was identified as a matrix-associated region (MAR), an AT-rich sequence that mediates attachment to the nuclear matrix (115). In transgenic experiments, L2a was found to have negative transcriptional activity using an E8_I_/E8_V_ hCD2 reporter construct that was reversed by the MAR binding protein, Satb1 (special AT-rich sequence binding protein 1) (116). Deletion at the endogenous locus as well as assays for position and orientation independence will help clarify whether this is a bona fide silencer. Thus, in contrast to the *Cd4* locus, there is currently no known negative element that confers helper versus cytotoxic lineage specificity of CD8 expression. One ontological explanation is that there may be a need for greater plasticity in CD8 expression compared to CD4. For example, positive selection of DP thymocytes induces the downregulation of CD8, which is reversed during cytotoxic lineage commitment (7). The overexpression of Thpok in CD8^+^ cytotoxic T cells silences CD8 but does not induce CD4 expression (8, 117). *In vivo*, some CD4^+^ helper T cells in the intestine undergo transcriptional reprogramming toward a cytotoxic phenotype including upregulation of CD8αα following the downregulation of Thpok (118, 119). Thus, dynamic and flexible CD8 expression in T cells may require regulation mediated directly through a transcriptional circuit.

### Epigenetic Regulation of CD8 Expression

The variegated CD8 expression in DP thymocytes due to deletion of E8_I_/_II_ or E8_V_ suggested that these enhancers were required to protect the *Cd8* locus from repressive chromatin modifications. Interestingly, mutations in the SWI/SNF-like nucleosome remodeling BAF complex caused similar CD8 variegation in DP thymocytes as the enhancer-deficient mice (82, 110, 112). BAF likely regulates *Cd8* directly, as it was found to bind to the locus by ChIP analysis (82). The DNA-binding transcriptional regulator Ikaros, which was reported to associate with the BAF complex, was also required for efficient CD8 upregulation during the DN–DP transition when on an Aiolos-deficient background (another Ikaros family member) (120, 121). In T cells, Ikaros was also found to associate with the repressive chromatin remodeling complex NuRD (122). The NuRD ATPase chromatin remodeling subunit, mi-2β, was found to bind the *Cd8* locus until pre-TCR signaling, upon which its eviction coincided with increased DNAse I hypersensitivity and expression of CD8 (123). Thus, different chromatin remodeling complexes, presumably recruited by transcription factors such as Ikaros to the CD8 enhancers, can activate or repress CD8 expression at different stages of development.

Double-positive thymocytes that failed to upregulate CD8 in E8_I/II_-deficient mice showed decreased histone acetylation at the *Cd8* locus concomitantly with a decrease in H3K4Me3, both consistent with the loss of expression (124). H3Ac was also decreased along with an increase in H3K27Me3 in activated cytotoxic T cells from E8_I_^−/−^ mice that had lost CD8 expression (109). Treatment of E8_I_^−/−^ CD8^+^ T cells with the HDAC inhibitor TSA partially rescued CD8 expression during activation although there was no restoration of CD8 in T cells that had already lost expression (109). Interestingly, the unstable CD8 expression during proliferation was reminiscent of CD4 expression in the absence of either E4_M_ or E4_P_ (73, 75).

Histone acetylation appears to play a particularly prominent role in CD8 regulation, as the deletion of HDAC1/2 with CD4-Cre caused CD8 derepression in CD4^+^ T cells (125). In contrast, Dnmt1 deletion did not affect CD8 expression in CD4^+^ T cells but caused CD8 derepression in TCRγδ cells, suggesting differential dependence on DNA methylation and histone modifications for CD8 silencing in different cell types (126). DNA methylation was also analyzed in thymocytes that had failed to upregulate CD8 during the DN–DP transition in E8_I_/_II_-deficient mice (124). Previous studies showed that DNA methylation patterns correlate with CD8 expression during thymocyte development and in peripheral T cells (126–129). However, in the absence of E8_I_/_II_, there was a gain in methylation of several CpGs at the *Cd8* locus at E8_V_, and the expression of CD8 could be partially rescued by crossing the mice onto a Dnmt1-deficient background (124). These results suggest that factors binding to E8_I_/_II_ promote *Cd8* expression through inhibition of the DNA methylation machinery.

### Key Regulators of the *Cd4* and *Cd8* Epigenetic Landscape

#### Mazr

The BTB-ZF family member Mazr was identified in a yeast-one-hybrid screen to bind to E8_II_ (124). By ChIP-PCR, it was shown that Mazr also binds at numerous locations in the *Cd8* locus at the DN stage, and this binding is reduced in DP thymocytes following CD8 upregulation. Ectopic expression of Mazr-induced variegated CD8 expression in DP thymocytes, while deletion of Mazr on an E8_I_/E8_II_-deficient background reduced CD8 variegation (124, 130). However, in WT cells, Mazr deficiency was insufficient to derepress CD8 expression in DN thymocytes, suggesting redundancy with other repressive factors or lack of activating factors to initiate expression. Mechanistically, Mazr was found to bind to the co-repressor N-CoR, a component of large repressor complexes that include HDACs, and presumably Mazr can recruit these complexes to silence CD8 in DN thymocytes. Mazr was also connected to the network of transcription factors that regulate lineage commitment through the discovery of its role in the regulation of the T helper commitment factor Thpok (130). In the absence of Mazr, *Thpok* was derepressed in DP thymocytes, leading to lineage redirection of MHC class I selected thymocytes into the helper lineage. The derepression of *Thpok* was enhanced when combined with mutations in *Runx1* or *Runx3* (131). CD4 derepression was also enhanced in DN thymocytes doubly deficient for Runx proteins and Mazr, supporting a cooperative role between these factors (131). Mazr likely regulates Thpok expression directly as it was found to bind the *Thpok* silencer element (130). Mazr-mediated silencing of *Thpok* is also important in the periphery as Mazr deficiency inhibited the induction of cytotoxic CD4^+^ T cells in the intestine through the inability to fully downregulate Thpok (118).

#### Thpok

The key transcription factor that directs commitment to the CD4^+^ lineage is Thpok, also known as Zbtb7b or cKrox. A role for Thpok in thymocyte development was discovered after the identification of a spontaneous mutant mouse strain deficient in T helper cells, referred to as helper-deficient (HD) mice (132, 133). The mutation was mapped to an amino acid substitution in the second zinc finger domain of Thpok, and the causal role of the mutation in the HD phenotype was confirmed by transgenic rescue of CD4^+^ lineage development with WT Thpok (133, 134). Furthermore, the HD mouse strain could not be rescued by an MHC class II restricted TCR transgene, and there was no evidence of impaired positive selection in the thymus, indicating that *Thpok* was downstream of TCR signaling. Another study investigated *Thpok* based on its significant upregulation following MHC class II selection in the thymus (134). Ectopic expression of *Thpok* redirected MHC class I selected thymocytes to the helper lineage, indicating it was both required and (in the context of expression of other factors, such as GATA3) sufficient for CD4^+^ lineage commitment (133, 134).

Mechanistically, Thpok plays a critical role in lineage commitment through its antagonism of the CD8^+^ lineage transcription factor Runx3. Thpok was found by ChIP to bind to the *Cd4* silencer in CD4SP cells, and ectopic Thpok expression in DN thymocytes caused CD4 derepression, suggesting that Thpok directly antagonizes Runx activity at the silencer (92, 135). Thpok may continue to antagonize CD4 silencing in mature T cells, as peripheral deletion of *Thpok* led to aberrant populations of MHC class II restricted T cells that were CD4^lo^CD8^−^ and CD4^+^CD8^+^. These populations were reduced by the additional absence of Runx complexes, suggesting that postthymic Thpok is required for both the proper expression of CD4 and the repression of CD8 in helper T cells (119, 136). However, inducible inactivation of *Thpok* in peripheral T cells as well as the physiological downregulation of *Thpok* in the intestine did not produce a population of CD4^−^/CD4^lo^ helper T cells (118, 137). This may be due to the leakiness of the transgenes used to drive *Thpok* excision in the different studies or technical limitations of inducible systems, and further studies are warranted to determine whether Thpok is needed for CD4 expression postthymically. Finally, analysis of mice with GFP inserted into the *Thpok* locus and YFP inserted into the *Runx3* locus demonstrated that Thpok was required to repress Runx3 expression in MHC class II selected cells (138). Conversely, helper T cell differentiation in the absence of Thpok could be rescued by Runx deficiency (138). Taken together, these data suggest a mutual antagonism between these critical transcription factors for CD4^+^ and CD8^+^ T cell differentiation.

The above studies also suggested that, in contrast to the epigenetic silencing of CD4, CD8 silencing in mature CD4 lineage cells depends on the continuous activity of Thpok. As has been reported for other BTB-ZF family members, Thpok function has been linked to repression through HDAC activity (135, 139). At the *Cd8* locus, Thpok has been found to occupy the CD8 enhancers and promoter in CD4SP thymocytes (139). The HDAC inhibitor TSA blocked the repressive activity of Thpok in luciferase assays, and numerous HDACs were found to associate with Thpok by Co-IP (139). Interestingly, a mutant form of Thpok that prevented its interaction with Hdac4 was unable to inhibit CD8 expression when overexpressed in CD8^+^ T cells (117, 139). Although the mutation did not affect Thpok binding to the *Cd8* locus, it abrogated recruitment of Hdac4. In addition, in a CD4^+^ thymoma cell line, Thpok was unable to inhibit Runx-mediated repression of an E4_p_-S4 GFP reporter construct after TSA treatment (135). Thus, HDACs seem to be essential components for Thpok function, but additional *in vivo* studies of their complementary roles are needed.

**Figure 6:**
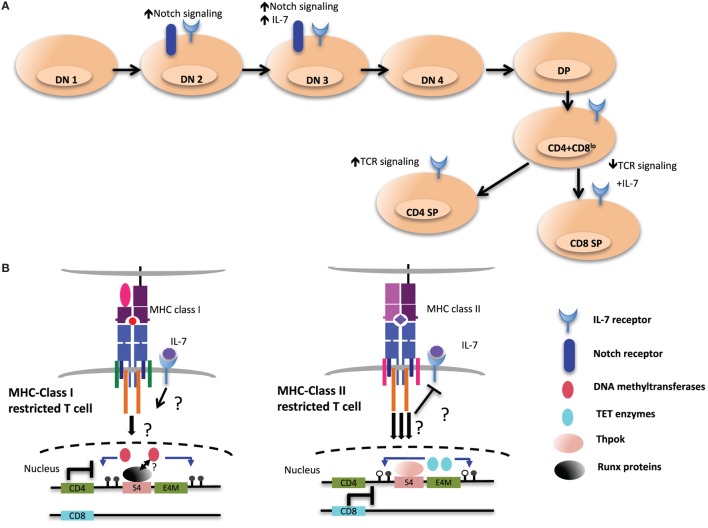
**Environmental cues during T cell development may shape the epigenetic landscape of CD4 and CD8 T cells**. **(A)** Extracellular signaling from the epithelial stroma is a key to guide the multiple stages of αβ T cell development in the thymus. Notch signaling during the early double-negative (DN) stages of T cell development inhibits potential for other lineages, such as B cell and myeloid cells. After thymocytes successfully pass β-selection, Notch signaling is turned off as a consequence of pre-T cell receptor signaling, and subsequent stages of T cell development exhibit very low levels of Notch signaling. Meanwhile IL-7 signaling at this particular stage is critical for the survival and the proliferation of β-selected cells. IL-7 signaling is terminated in immature CD4^+^CD8^+^ double-positive (DP) thymocytes through downregulation of receptor, which is re-expressed in postpositive selection CD4^+^CD8^lo^ intermediate cells, which not only provides prosurvival signals but also cues for distinct T cell lineage differentiation. **(B)** Persistent T cell antigen receptor (TCR) signaling, which has been proposed to desensitize IL-7 signaling and to favor CD4 lineage differentiation, could be a critical environmental cue to instigate the expression and activity of ten eleven translocation (TET) enzymes at the *Cd4* locus (right). In contrast, cessation of TCR signaling would allow IL-7R signaling in intermediate cells. This may contribute to CD8 lineage specification and may be a key signal to maintain the activity of DNA methyltransferases at the *Cd4* locus and ensure methylation of the locus (left).

Interestingly, initiation of demethylation of the *Cd4* locus coincides with the upregulation of Thpok, which is first detected in postselection CD4^+^CD8^lo^ thymocytes (100, 138). CD8^+^ T cells from *Thpok^GFP/^*^−^ mice, which are MHC class II restricted cells redirected from the CD4SP lineage, express GFP, suggesting that MHC class II restricted positive selection is required and sufficient to turn on high Thpok expression (138). However, the *Cd4* locus was still hypomethylated in MHC class II-selected cells redirected to the CD8^+^ lineage in the absence of Thpok compared to WT CD8^+^ lineage cells, suggesting that Thpok is partially dispensable for DNA demethylation. Therefore, additional unknown factors triggered during MHC class II-mediated TCR signaling may be responsible for coordinating TET-mediated demethylation of the locus (Figure 6).

Given the sufficiency and requirement of *Thpok* in T helper cell differentiation, a series of studies were undertaken to characterize the *cis* elements regulating its expression (92, 140). A genomic region upstream of the *Thpok* distal promoter, the distal regulatory element (DRE), was found to drive expression in the CD4^+^CD8^lo^ stage and in MHC class II selected cells. The DRE was subsequently dissected into two distinct elements, the *Thpok* silencer, which determined helper lineage specificity, and the thymic enhancer (TE), which drove expression early after positive selection (141). Interestingly, *Thpok* silencer deletion in mature CD8^+^ T cells did not cause derepression, indicating that *Thpok* was heritably silenced in cytotoxic T cells after thymic development, similar to the *Cd4* locus (87, 142). DHS studies also revealed the presence of another region in the *Thpok* locus that lies 1.8 kb downstream of the proximal promoter of *Thpok*. Termed the proximal regulatory element (PRE), this region was more accessible in CD4 lineage cells. An enhancer within the PRE, referred to as the PE, was essential for efficient *Thpok* induction after MHC class II selection (92). Deletion of either the PE or the TE within the *Thpok* locus led to redirection of MHC class II-selected cells toward the cytotoxic lineage (92, 141). The general T lymphoid element (GTE), a region between the DRE and PRE, was found to direct reporter expression in both helper and cytotoxic T cell lineages in transgenic mice (140). Thus, *Thpok* expression is regulated by at least three enhancers and one silencer element. Several Runx motifs were found within the *Thpok* silencer, and their deletion in the germline revealed critical roles for Runx complexes in silencing *Thpok* (140, 143). Furthermore, mice with mutations in *Runx1* and *Runx3*, or in the obligate Runx binding partner *Cbfb*, showed *Thpok* derepression in preselection DP thymocytes and loss of the cytotoxic lineage. Interestingly, Runx complexes were bound to the *Thpok* silencer in both CD4^+^ and CD8^+^ T cells, suggesting that Runx localization to the silencer was insufficient to repress *Thpok* (143). Indeed, Thpok was also bound to its own silencer, where it may antagonize Runx-mediated repression in helper T cells, thereby acting in a positive feedback loop.

#### Runx

The Runx family is composed of three evolutionally conserved transcription factors, Runx1, Runx2, and Runx3, which have important developmental roles including hematopoiesis, osteogenesis, and neurogenesis (90). Mammalian Runx genes make use of two promoters, a distal promoter (P1) and a proximal promoter (P2), to generate different transcripts. Expression of Runx1 and Runx3 in T cells is driven by distal P1-promoter activity (138, 144). Structurally, Runx proteins contain two conserved domains, an *N*-terminal DNA-binding Runt domain and the *C*-terminal VWRPY penta-peptide motif (145). The Runt domain binds to core-binding subunit-β (Cbfb) that is required for stabilizing the interaction of Runx proteins with DNA. The VWRPY motif is thought to act as a docking module for the Groucho/TLE co-repressor protein family, which is required for Runx-mediated repression of multiple genes. As discussed above, Runx1 and Runx3 are critical for CD4 silencing at the DN and CD8SP stages, respectively. Interestingly, silencing activity of Runx3 at the *Cd4* locus depends on the VWRPY motif, and mice expressing mutant Runx1 and Runx3 lacking this motif showed complete CD4 derepression in CD8^+^ T cells (146, 147). In contrast, the *Thpok* silencer remained partly functional in the absence of both Runx VWRPY motifs, suggesting that these factors employ different modes of transcriptional regulation of *Cd4* at different stages of development (147). Consistent with this observation, VWRPY-mediated recruitment of the co-repressor TLE2 was enriched at S4 relative to the *Thpok* silencer (147). VWRPY-independent silencing might involve Runx association with other repressive complexes. For instance, Runx proteins have been shown to associate with HDACs and Sin3A and thymic deletion of Sin3a in mice impairs the development of CD8^+^ T cells, reminiscent of Runx-deficient mice (143, 148–150). In addition to the Groucho and TLE co-repressor complexes, Runx1 was shown to interact with HDACs and SUV39H1 to repress transcription by way of a domain distinct from the VWRPY penta-peptide motif (151). However, whether this association plays a role in the epigenetic silencing of *Cd4* or lineage commitment is unknown.

Although S4 is required for the proper methylation of the *Cd4* locus in CD8^+^ T cells, it is presently unclear whether Runx3 plays a role in mediating methylation changes in CD8^+^ T cells. As the selection of CD8^+^ T cells has been proposed to be promoted by downregulation of CD8 expression at the CD4^+^CD8^lo^ stage, due to transient reduction of avidity of TCR interaction with MHC class I complexes (7), it is tempting to speculate that TET enzymes are not recruited to the *Cd4* locus in CD8^+^ T cells as a result of reduced TCR signaling. Alternatively, IL-7R signaling in MHC class I selected T cells may inhibit TET enzyme recruitment/activity by way of yet to be determined factors (Figure 6).

Runx complexes are also important for promoting CD8 expression during T cell development. ChIP experiments have revealed binding of Runx complexes to E8_I_, E8_II_, and E8_IV_ in CD8SP and DP thymocytes (94). Runx1 deficiency reduced CD8 expression in DP thymocytes, while Runx3 deficiency reduced expression in CD8SP thymocytes (91, 152). In the absence of Runx3 or Cbfb, CD8 expression was also unstable in peripheral CD8^+^ T cells activated *ex vivo* (109). However, inactivation of Runx complexes in mature CD8^+^ T cells did not lead to loss of CD8 expression, suggesting that Runx complexes prime the *Cd8* locus through enhancer activity during development, enabling heritable activation in peripheral T cells. The mechanisms by which Runx proteins mediate the heritable activation of CD8 expression in mature cytotoxic T cells and coordinate deposition of active chromatin marks is not known. In addition to *Cd4* silencing and *Cd8* activation, Runx proteins have multiple other important roles in T cell development. Deletion of Runx1 impaired β-selection, positive selection, and the survival of the helper lineage, while Runx3 has been shown to be essential for efficient CD8^+^ T cell differentiation by repressing CD4^+^ lineage genes such as *Thpok* and activating cytotoxic lineage genes such as those encoding perforin, granzyme B, and CD103 (143, 146, 152, 153). However, ectopic expression of Runx3 was insufficient to induce CD8^+^ lineage redirection, suggesting that it may be epistatic to other factors induced by MHC class II selection (11, 143).

#### Tcf1 and Lef1

T cell factor 1 and Lef1 (encoded by *Tcf7* and *Lef1* genes, respectively) are HMG transcription factors of the Tcf/Lef family that control key steps in development during T cell maturation (154). Notch signaling in early thymic progenitors induces expression of Tcf1, which then employs various mechanisms to ensure T cell lineage commitment, including promoting β-selection at the CD4^−^CD8^−^ double-negative 3 stage and preventing malignant transformation (Figure 6, top) (155, 156). It was recently shown that Tcf1 and Lef1 are required for CD4^+^ lineage commitment, and a deficiency of Tcf1 and Lef1 results in lineage redirection of MHC class II selected cells into CD8^+^ T cells (157). This is in part mediated by direct regulation of *Thpok* as Tcf1 binds the GTE in the *Thpok* locus (140). In agreement with Tcf1 acting upstream of *Thpok*, ectopic expression of *Thpok* rescued the CD4^+^ T cell defect in *Tcf7*^−^*^/^*^−^*Lef1*^−^*^/^*^−^ mice. Notably, the expression of additional transcription factors important for CD4^+^ T cell differentiation, GATA3, Myb, and Tox, was unchanged in the absence of Tcf1 and Lef1 (157).

*Tcf7*^−^*^/^*^−^*Lef1*^−^*^/^*^−^ mice also showed derepression of CD4 in CD8SP cells, without changes in Runx3 expression. Tcf1 co-immunoprecipitated with Runx3, and ChIP analysis showed binding to S4. Mechanistically, Tcf1 likely cooperates with Runx complexes to silence CD4 as loss of Runx3 together with Tcf1 and Lef1 led to increased derepression of *Cd4*. Although Lef/Tcf members are known to interact with Groucho/TLE, physical interaction between Tcf1 and Runx3 was shown to occur independently of Groucho-TLE (158–160). Interestingly, an intrinsic HDAC domain in Tcf1 and Lef1 was required for the repression of genes associated with the CD4^+^ lineage, including *Cd4, Cd40lg, FoxP3*, and *Rorc* in CD8SP cells (158). Purified Tcf1 caused deacetylation of both H3K9Ac and H3K27Ac protein substrates *in vitro*, and this activity was abrogated in mutants lacking the putative 30 amino acid HDAC domain. *In vivo*, about 80% of Tcf1 target genes in CD8SP cells had elevated H3K27Ac and H3K9Ac in the absence of Tcf1 and Lef1. However, such marks were also found in a proportion of upregulated genes that did not harbor Tcf1-binding sites, suggesting indirect changes in acetylation as well. Furthermore, association with canonical HDACs could be responsible for deacetylation of certain genes, as physical interaction of Lef1 with HDAC1 was previously demonstrated (161). Tcf1-HDAC activity may also modify non-histone substrates. As Runx3 was found acetylated in some cancers, Tcf1 may act directly on Runx3 to modulate CD4 silencing (162, 163). It will be interesting to determine how Tcf1 and Lef1 HDAC activity is regulated, particularly with regards to target genes it activates, as deacetylation is typically associated with silencing.

## Perspectives and Future Directions

In this review, we have emphasized how the studies of *Cd4* and *Cd8* regulation have revealed key insights into epigenetic mechanisms that mediate lineage commitment and maintain gene expression patterns that determine cell identity. The dynamic expression pattern of the co-receptor genes, from transient expression in DP thymocytes to stable expression or repression in CD4SP and CD8SP cells, provides a tractable system to understand mechanisms of heritability. Although *cis* elements are required for transcriptional control of *Cd4*, they are also required to direct epigenetic marks essential for heritable transmission in a stage-dependent manner. Recent discoveries have highlighted a key role for these elements in modulating DNA methylation changes that are key for heritable CD4 expression and histone acetylation in controlling dynamic CD8 expression. Excitingly, these studies have opened doors to understanding how antagonistic epigenetic processes can be co-regulated. For instance, it remains unclear how S4 inhibits DNA demethylation of the *Cd4* locus while entraining the Dnmt enzymes in CD8^+^ T cells and how the *Cd4 cis* elements direct demethylation through the TET enzymes. The mechanisms regulating HDAC activity at the *Cd8* locus for silencing expression in the CD4^+^ lineage also warrants additional study. Moreover, other branches shaping the epigenetic landscape have yet to be explored. Although the lncRNA transcriptome across bone marrow and thymic progenitors has been sequenced in humans, the functional contribution of these and other ncRNAs in T cell lineage commitment remains largely unexplored (164). Thus, it will be interesting to examine how lncRNA, enhancer RNA, microRNA, and other ncRNA species participate in lineage commitment and modulate chromatin at lineage-specific gene loci such as *Cd4, Cd8*, and *Thpok*. Furthermore, DNA demethylation at the *Cd8* locus and its relationship with the CD8 *cis* elements is yet to be determined. The contribution of histone variants and their chaperones to gene expression is also likely to advance our understanding of lineage commitment, as is more detailed mapping of chromatin interactions between the different *cis* elements at the *Cd4* and *Cd8* loci. With advances in technology such as CRISPR/Cas9 genome editing, single-cell sequencing and locus-specific manipulation of chromatin, the pieces of the puzzle of heritability of gene expression can now begin to be assembled.

## Author Contributions

All authors listed have made substantial, direct, and intellectual contribution to the work and approved it for publication.

## Conflict of Interest Statement

The authors declare that the research was conducted in the absence of any commercial or financial relationships that could be construed as a potential conflict of interest.

## Abbreviations

E4M, maturity enhancer; E4P, proximal enhancer; S4, silencer; E4D, distal enhancer; E4T, thymocyte enhancer; Thpok, T helper inducing POZ/Krueppel-like factor; DNMT, DNA methyltransferase; MDB, methyl-CpG-binding domain; TET, ten eleven translocation; 5mC, 5-methyl cytosine; 5hC, 5-hydroxymethyl cytosine; HAT, histone acetyltransferase; HDAC, histone deacetylase enzyme; HP1, heterochromatin protein 1; DP, double positive; DN, double negative; CD4 SP, CD4 single positive; CD8 SP, CD8 single positive; Tcf1, T cell factor 1; Lef1, lymphoid enhancer factor 1; BAF, Brg/Brahma-associated factors; Uhrf1, ubiquitin like with PHD and ring finger domains 1; NuRD, nucleosome remodeling and histone deacetylase; AP4, adaptor-related protein complex 4.
